# Supplementary material for: Absence of HDAC3 by Matrix Stiffness Promotes Chromatin Remodeling and Fibroblast Activation in Idiopathic Pulmonary Fibrosis
Source: Cells. 2023 Mar 27;12(7):1020. doi: 10.3390/cells12071020 (PMC10093275; doi:10.3390/cells12071020)
Supplement: Supplementary file 1 [file cells-12-01020-s001.zip › cells-2207322-supplementary.pdf]

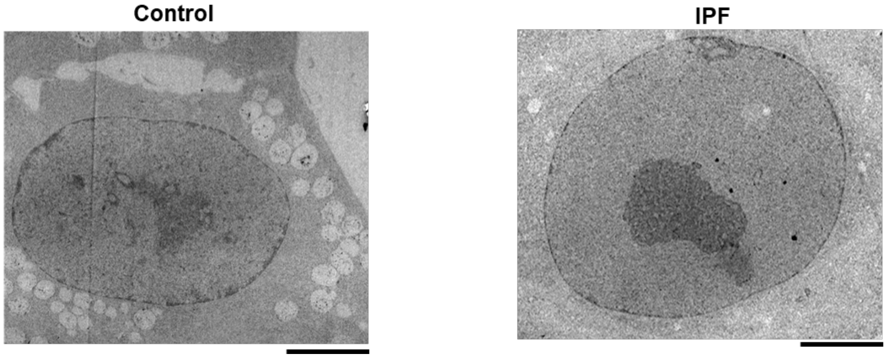

Figure S1. Increased nuclear are in IPF by transmission electron microscopy. a Representative transmission electron microscopy of control and fibroblast nuclei. Scale bar: 5μm.

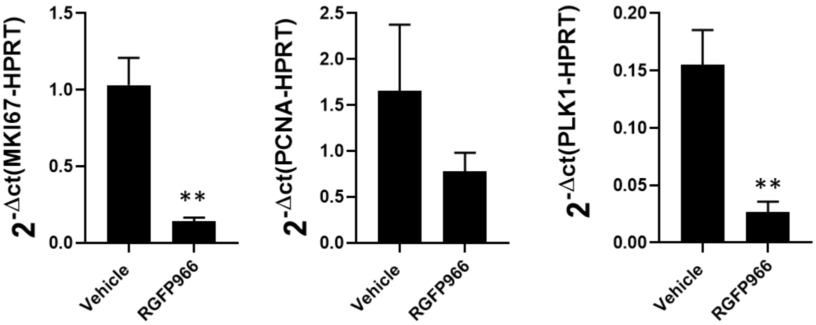

Figure S2. Pharmacological inhibition of HDAC3 by RGFP966 regulates the expression of cell proliferation genes. qPCR analysis of fibrosis-related genes: MKi67, PCNA, and PLK1, control fibroblasts treated with RGFP966 10μM for 24 hours from three independent experiments. **P < 0.01 by unpaired t-student test.

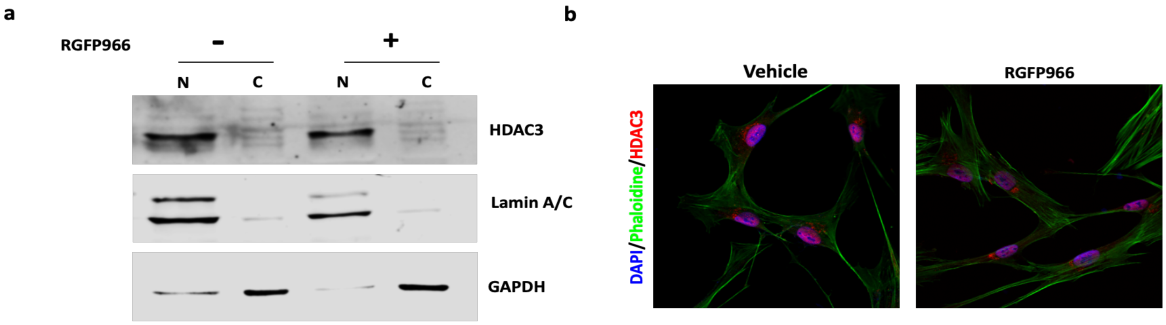

Figure S3. Absence of nuclear HDAC3 translocation after RGFP966 treatment. (a) Representative immunoblots of HDAC3 from nuclear and cytoplasmic extracts of fibroblasts treated or not with RGFP966 (1μM) for 24 hours. **(b)** Immunofluorescence images of HDAC3 in control fibroblast non-treated and treated with RGFP966, HDAC3 (red), phalloidin (green), and DAPI (blue).
